# BcMF30a and BcMF30c, Two Novel Non-Tandem CCCH Zinc-Finger Proteins, Function in Pollen Development and Pollen Germination in *Brassica campestris* ssp. *chinensis*

**DOI:** 10.3390/ijms21176428

**Published:** 2020-09-03

**Authors:** Liai Xu, Xingpeng Xiong, Weimiao Liu, Tingting Liu, Youjian Yu, Jiashu Cao

**Affiliations:** 1 Laboratory of Cell & Molecular Biology, Institute of Vegetable Science, Zhejiang University, Hangzhou 310058, China; 11416052@zju.edu.cn (L.X.); xiongxingpeng1989@163.com (X.X.); 11616044@zju.edu.cn (W.L.); 11416009@zju.edu.cn (T.L.); 2 Key Laboratory of Horticultural Plant Growth, Development and Quality Improvement, Ministry of Agriculture/Zhejiang Provincial Key Laboratory of Horticultural Plant Integrative Biology, Hangzhou 310058, China; 3 Department of Horticulture, College of Agriculture and Food Science, Zhejiang A & F University, Lin’an 311300, China; yjyu@zafu.edu.cn

**Keywords:** CCCH zinc-finger protein, *Brassica campestris* ssp. *chinensis*, pollen development, pollen germination, male fertility

## Abstract

Chinese cabbage (*Brassica campestris*) is an economically important leaf vegetable crop worldwide. Mounting studies have shown that cysteine-cysteine-cysteine-histidine (CCCH) zinc-finger protein genes are involved in various plant growth and development processes. However, research on the involvement of these genes in male reproductive development is still in its infancy. Here, we identified 11 male fertility-related CCCH genes in Chinese cabbage. Among them, a pair of paralogs encoding novel non-tandem CCCH zinc-finger proteins, *Brassica campestris Male Fertility 30a* (*BcMF30a*) and *BcMF30c*, were further characterized. They were highly expressed in pollen during microgametogenesis and continued to express in germinated pollen. Further analyses demonstrated that both BcMF30a and BcMF30c may play a dual role as transcription factors and RNA-binding proteins in plant cells. Functional analysis showed that partial *bcmf30a bcmf30c* pollen grains were aborted due to the degradation of pollen inclusion at the microgametogenesis phase, and the germination rate of viable pollen was also greatly reduced, indicating that *BcMF30a* and *BcMF30c* are required for both pollen development and pollen germination. This research provided insights into the function of CCCH proteins in regulating male reproductive development and laid a theoretical basis for hybrid breeding of Chinese cabbage.

## 1. Introduction

The eukaryotic genome encodes a large number of proteins containing a variety of zinc-finger motifs and forms one of the most abundant protein families in eukaryotes. Based on the arrangements of cysteine (C) and/or histidine (H) residues in the zinc-finger motif that bound to zinc ions, zinc-finger proteins can be classified into several different types, including C_4_, C_6_, C_8_, C_2_H_2_, C_2_HC, C_2_HC_5_, CCCH, C_3_HC_4_, and C_4_HC_3_ [1,2]. These proteins are known to involve in numerous cellular functions (e.g., DNA recognition, RNA binding, transactivation, and protein-protein interaction) through their conserved zinc-finger motifs [3,4]. Cysteine-cysteine-cysteine-histidine (CCCH) zinc-finger proteins usually possess one to seven copies of CCCH motifs, which are newly defined as C-X_4–17_-C-X_4–6_-C-X_3_-H [5,6,7]. CCCH genes are widely distributed in organisms from bacteria to eukaryotes [5,8], and members of this gene family in various plants have also been predicted in silico [5,6,7,9,10,11,12]. For instance, 68 CCCH-type zinc-finger protein genes divided into 11 subfamilies have been identified in the *Arabidopsis thaliana* genome [5]. The majority of these proteins contain one or two CCCH motifs. Notably, there are 11 members contain a tandem CCCH zinc-finger (TZF) motif preceded by a conserved arginine-rich (RR) region, known as AtTZF1–AtTZF11 [13,14,15].

In recent years, increasing reports have continuously demonstrated that CCCH genes play pivotal roles at different stages of plant growth and development. *AtTZF6*/*PEI1* is the first CCCH gene to be functionally studied in plants, which is participated in embryo development [16]. *AtTZF1* affects sugar-, ABA-, and GA-mediated growth [17]. *AtTZF4*/*SOMNUS*, *AtTZF5*, and *AtTZF6*/*PEI1* play roles in seed germination [18,19]. *AtC3H14* and *AtC3H15*/*CALLOSE DEFECTIVE MICROSPORE 1* (*CDM1*) are involved in the regulation of secondary wall thickening and male fertility [20,21]. In rice (*Oryza sativa*), *Delay of the Onset of Senescence* (*OsDOS*), *OsTZF1*, *GluB-1-binding Zinc Finger 1* (*OsGZF1*), *ILA1 Interacting Protein 4* (*OsIIP4*), and *Defective Callose in Meiosis 1* (*DCM1*) all function in plant development [22,23,24,25,26]. The CCCH proteins are also important regulators of stress responses. Several *AtTZFs* are shown to be triggered by various stimuli including salt stress, extreme temperatures, drought, nutritional deficiency, and hormone treatment [5,18,27]. Furthermore, the overexpression of these genes usually can enhance abiotic stress tolerance [17,23,27,28]. *AtTZF9* is the only known RR-TZF gene that regulates immunity in Arabidopsis [29,30].

Nonetheless, CCCH proteins that have been functionally characterized mainly focus on model plants, Arabidopsis, and rice, and most of them are TZF proteins. To date, only a few CCCH genes have been characterized in other plants. For instance, the overexpression of cotton (*Gossypium hirsutum*) *Zinc Finger Protein 1* (*GhZFP1*) can enhance tolerance to salt stress and resistance to fungal disease [31]. *GhTZF1* is involved in drought stress responses and leaf senescence [32]. The pepper (*Capsicum annuum* L.) TZF CaC3H14 acts as a positive regulator in response to *Ralstonia solanacearum* [33]. In poplar, two homologues of *AtC3H14*, *PdC3H17* and *PdC3H18*, can also regulate secondary xylem formation [34]. For non-TZF proteins, functional studies are also very limited, even in model plants. HUA1, the only protein in Arabidopsis that contains six CCCH motifs, has been characterized as an RNA-binding protein and is involved in flower morphogenesis [35,36]. *AtC3H17* plays multiple roles in plant development and stress response [37,38]. *KHZ1* and *KHZ2* could mediate flowering and senescence in Arabidopsis [39,40]. DCM1 is a five CCCH motif-containing protein in rice that is involved in male meiotic cytokinesis [26]. IbC3H18 is a nuclear transcriptional activator that can enhance abiotic stress tolerance in sweet potato (*Ipomoea batatas* L.) [41]. Therefore, it seems that non-TZF proteins also play vital roles in plant growth and development, as well as stress response.

Chinese cabbage (*Brassica campestris* L. ssp. *chinensis* Makino) is a subspecies of *B. campestris* (AA, 2*n* = 20) and one of the most important vegetable crops in Asia. In the breeding of Chinese cabbage, due to its strong heterosis characteristics, male sterile plants have been widely used in the production of hybrid seeds. The identification and functional analysis of genes related to male fertility is the basis of constructing male sterile lines. Although there is mounting evidence that CCCH genes are involved in various plant developments, only a handful of genes mentioned above have been characterized to play roles in male fertility. More than 100 CCCH genes have been identified in silico in Chinese cabbage [11,12]. However, as far as we know, no function of any of these genes has been revealed, suggesting that their roles in male fertility are yet to be ascertained in Chinese cabbage.

Therefore, in this study, we screened several male fertility-related CCCH zinc-finger protein genes by identifying the differentially expressed genes between the ‘*Bcajh97-01A/B*’ genetic male sterile (GMS) A/B lines of *B.campestris*. Among them, both *Brassica campestris Male Fertility 30a* (*BcMF30a*) and its paralog *BcMF30c* encode novel non-TZF proteins. The spatiotemporal expression profiles manifested that both of them were mainly expressed in developing pollen and geminated pollen. Further analyses demonstrated that both BcMF30a and BcMF30c have transcription activation activity in yeast and exhibit a dual nuclear and cytoplasmic localization; they contain RNA-binding domains as well, suggesting that they may play a dual role as transcription factors and RNA-binding proteins in plant cells. Moreover, we showed that mutation in *BcMF30a* and *BcMF30c* can cause abnormal pollen development, leading to pollen abortion and a failure of pollen germination. Together, our results indicate for the first time that two novel non-TZF proteins play key roles during microgametogenesis and pollen germination in Chinese cabbage.

## 2. Results

### 2.1. Identification of Male Fertility-Related CCCH Zinc-Finger Protein Genes in B. campestris

**Figure 1:**
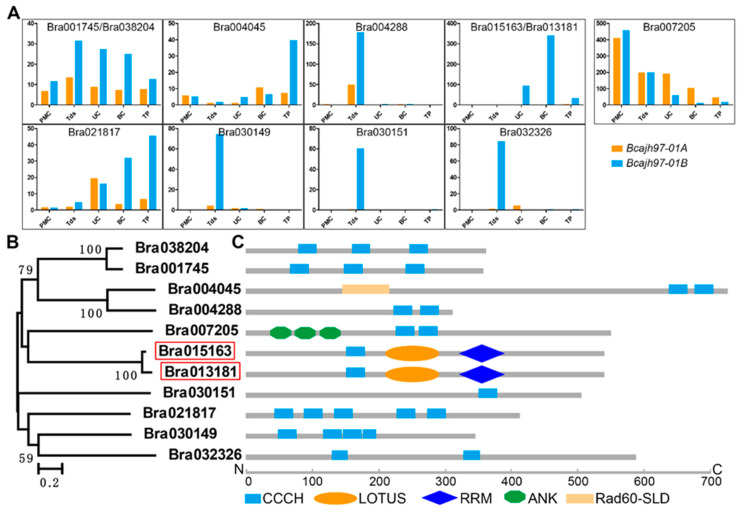
Identification of male fertility-related CCCH zinc-finger protein genes in *B. campestris*. (**A**) Expression analysis of CCCH zinc-finger protein genes in five pollen developmental stages of ‘*Bcajh97-01A/B*’ by RNA-seq. PMC, pollen mother cell stage; Tds, tetrads stage; UC, unicellular pollen stage; BC, bicellular pollen stage; TP, tricellular pollen stage. (**B**) Phylogenesis analysis of 11 male fertility-related CCCH zinc-finger proteins. (**C**) Distribution of the conserved domains of male fertility-related CCCH zinc-finger proteins.

In long-term study, we generated and cultivated a stable Chinese cabbage-pak-choi ‘Aijiaohuang’ (*Brassica campestris* L. ssp. *chinensis* Makino) GMS A/B line system, which is named ‘*Bcajh97-01A/B*’. The only difference between A/B lines is that the male sterile plant (‘*Bcajh97-01A*’) undergoes an aberrant meiotic cytokinesis at the microsporogenesis stage and thus could not produce any functional mature pollen [42,43]. Therefore, we used the RNA-seq data of ‘*Bcajh97-01A/B*’ to identify the male fertility-related CCCH genes in *B. campestris* [44]. When analyzing the expression of these genes in five pollen development stages of ‘*Bcajh97-01A/B*’, 11 genes were significantly differentially expressed in at least at one stage between the fertile plant ‘*Bcajh97-01B*’ and the sterile plant ‘*Bcajh97-01A*’, and all genes were downregulated in the sterile plant except Bra007205 (Figure 1A). Moreover, the expression patterns of these genes were also different. More precisely, Bra001745/Bra038204 were nearly uniformly expressed at five different stages of floral buds; Bra004045 and Bra021817 were highly expressed at the mature pollen stage; Bra004288, Bra030149, Bra030151, and Bra032326 were tetrad stage specifically expressed genes; Bra015163/Bra013181 were mainly expressed during the bicellular pollen stage; and Bra007205 was a highly expressed gene at the pollen mother cell stage.

Phylogenetic analysis showed that in addition to two pairs of paralogous genes (Bra001745/Bra038204 and Bra015163/Bra013181), Bra004045 and Bra004288 also have relatively close phylogenetic relationships (Figure 1B). The exon-intron structures of these 11 genes were also analyzed, and the number of exons varies greatly (Figure S1A). For example, Bra007205 has only 1 exon, while Bra032326 and Bra004045 have 11 and 12 exons, respectively. Protein domain analysis revealed that multiple proteins only contain CCCH motifs, ranging in number from 2 to 5. However, Bra004045 also contains a Rad60-SLD domain, Bra007205 contains three ANK motifs, and both Bra015163 and Bra013181 contain a Limkain, Oskar, and TUdor-containing proteins 5 and 7 (LOTUS) domain and an RNA recognition motif (RRM) domain in addition to CCCH motif(s) (Figure 1C). Further exploration showed that most of the CCCH motifs of these 11 proteins are C-X_8_-C-X_5_-C-X_3_-H type (Figure S1B). The syntenic gene analysis indicated that most of these genes (except Bra030149) have syntenic paralogs in *Arabidopsis thaliana*, and the corresponding encoded proteins contain the same number of CCCH motifs except Bra004045 (Table S1). Unsurprisingly, there are also few studies on the function of these homologous genes in male reproductive growth.

Together, we identified 11 CCCH zinc-finger protein genes that may be involved in male fertility in *B. campestris*. Although they are all expressed during pollen development, they display different expression patterns and differ in gene and protein structures.

### 2.2. Expression Patterns of Two Novel Non-TZF Protein Genes, BcMF30a and BcMF30c

Among the 11 male fertility-related genes identified above, two homologous genes, Bra015163 and Bra013181, encode proteins with only one CCCH motif, indicating that they are non-TZF protein genes. Bra015163 and Bra013181 are located in the less fractionized subgenome (LF) and the most fractionized subgenome (MF2), respectively, which means that the homologous gene in the moderate fractionized subgenome (MF1) has been lost. Thus, we named Bra015163 and Bra013181 as *BcMF30a* and *BcMF30c*, respectively. In addition to a CCCH motif, both BcMF30a and BcMF30c contain a LOTUS domain and an RRM domain (Figure 1C). Sequence analysis indicated that their amino acid similarity exceeded 90% (Figure S2).

**Figure 2:**
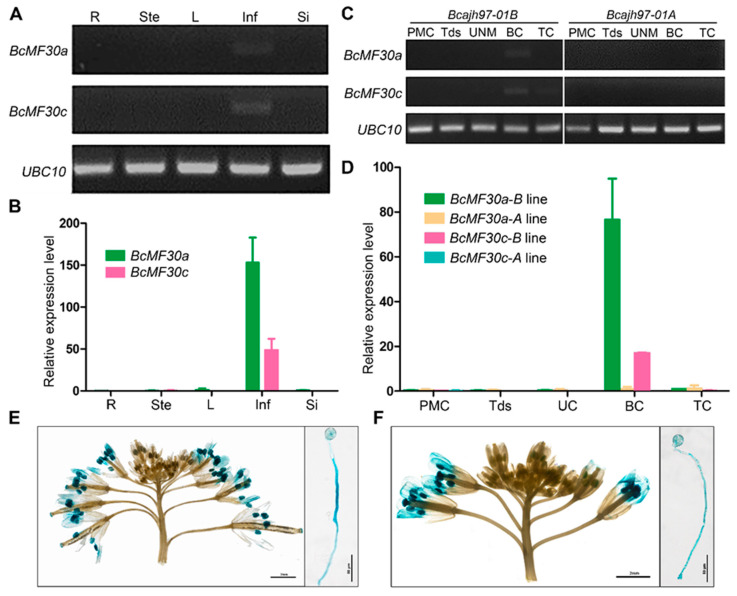
Expression analysis of *BcMF30a* and *BcMF30c*. Semi-RT-PCR (**A**) and qRT-PCR (**B**) analyses of *BcMF30a* and *BcMF30c* in five major tissues of ‘*Bcajh97-01B*’. R, root; Ste, stem; L, leaves; Inf, inflorescence; Si, silique. Semi-RT-PCR (**C**) and qRT-PCR (**D**) analyses of *BcMF30a* and *BcMF30c* in floral buds of ‘*Bcajh97-01A/B*’ at different developmental stages. PMC, pollen mother cell stage; Tds, tetrads stage; U, unicellular pollen stage; BC, bicellular pollen stage; TC, tricellular pollen stage. Ubiquitously expressed *UBC10* was used as internal control. Promoter-GUS activities of *BcMF30a* (**E**) and *BcMF30c* (**F**) in inflorescences and germinated pollen. Bars = 2 mm and 50 μm. GUS: β-glucuronidase.

To verify the effects of these two genes on Chinese cabbage, their spatiotemporal expressions were first analyzed with RT-PCR and qRT-PCR using total RNA extracted from various tissues. As shown in Figure 2A,B, both *BcMF30a* and *BcMF30c* transcripts only accumulated in inflorescence, and they were undetectable in root, stem, leaf, and silique. We also verified the expression of *BcMF30a* and *BcMF30c* in the floral buds at different developmental stages of ‘*Bcajh97-01A/B*’. Consistent with the expression profile based on RNA-seq analysis, they were mainly detected in ‘*Bcajh97-01B*’ floral buds at the bicellular pollen stage (Figure 2C,D). To investigate the expression of *BcMF30a* and *BcMF30c* in detail, we generated *ProBcMF30a:GUS* and *ProBcMF30c:GUS* transgenic Arabidopsis lines expressing the *GUS* (β-glucuronidase) reporter gene under the control of the promoters of *BcMF30a* and *BcMF30c*, respectively. Similar to the above results, GUS staining can be detected in flowers at late developmental stages both in *ProBcMF30a:GUS* and *ProBcMF30c:GUS* transgenic plants, and GUS signal was mainly in anthers. In vitro pollen germination analysis revealed that *ProBcMF30a:GUS* and *ProBcMF30c:GUS* were also expressed in germinated pollen and pollen tubes (Figure 2E,F). These results indicated that *BcMF30a* and *BcMF30c* are pollen and pollen tube specifically expressed genes.

### 2.3. Transcriptional-Activation Assay and Subcellular Localization of BcMF30a and BcMF30c

**Figure 3:**
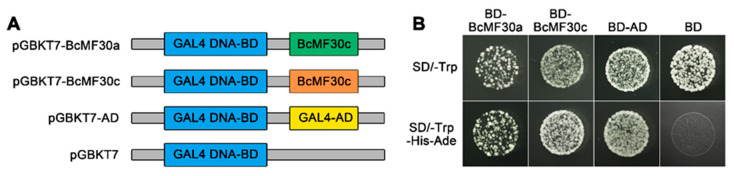
Transcriptional activation assays of BcMF30a and BcMF30c. (**A**) Schematic diagrams of plasmids used for transcriptional activation assays. GAL4 DNA-BD, GAL4 DNA-binding domain; GAL4-AD, GAL4 activation domain; BcMF30a and BcMF30c, the coding sequences of BcMF30a and BcMF30c. (**B**) The growth of transformed yeast on different yeast screening media.

Numerous zinc-finger proteins are known to bind to DNA through conserved zinc finger motifs and act as transcription factors to regulate the transcription of genes [3]. In order to verify whether BcMF30a and BcMF30c have transcript activation ability, yeast transactivation assay, a simple and quick method to test for transcriptional activation, was carried out. We fused the coding regions of *BcMF30a* and *BcMF30c* with the GAL4 DNA-binding domain in pGBKT7 vector (BD), respectively (Figure 3A). As shown in Figure 3B, the yeast cells harboring BD-AD, BD-BcMF30a, or BD-BcMF30c grew well on SD/-Trp-His-Ade medium, suggesting that both BcMF30a and BcMF30c possess transcriptional activation activity and might function as transcriptional activators, at least in yeast cells.

**Figure 4:**
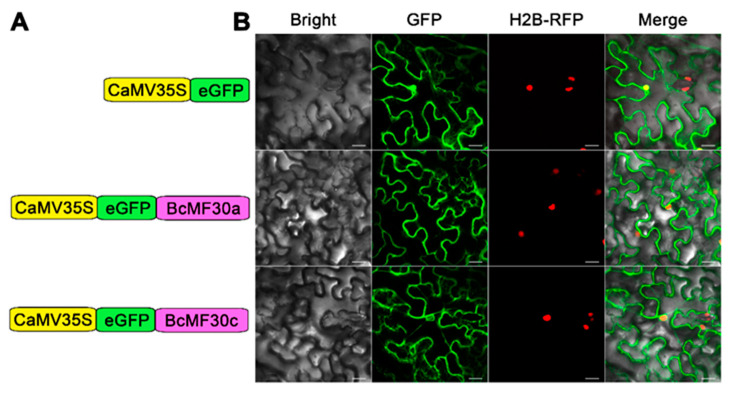
Subcellular localization of eGFP-BcMF30a and eGFP-BcMF30c fusion proteins in tobacco epidermal cells. (**A**) Constructs used for the subcellular localization analysis. (**B**) Both eGFP-BcMF30a and eGFP-BcMF30c were located in the nucleus and dispersed in the cytoplasm of tobacco epidermal cells. H2B is a marker protein of nucleus. Pictures represent white field images (Bright), epifluorescence (GFP and RFP) and merged images (Merge). Bar = 25 μm.

To test whether BcMF30a and BcMF30c are nuclear proteins and thus similar to most typical transcription factors, we constructed fusion proteins of *CaMV35S:eGFP-BcMF30a* and *CaMV35S:eGFP-BcMF30c* (Figure 4A), which were transiently expressed in tobacco epidermal cells of H2B-RFP (nuclear marker) transgenic plants via *Agrobacterium* injection methodology. Surprisingly, in addition to the weak signal in the nucleus, we also observed that the fluorescence signals of eGFP-BcMF30a and eGFP-BcMF30c were distributed throughout the cytoplasm (Figure 4B). Similar subcellular localization patterns were also observed when enhanced green fluorescent protein (eGFP) was translationally fused to the C-terminus of BcMF30a and BcMF30c (Figure S3). These results indicated that BcMF30a and BcMF30c may not only function as transcription factors in the nucleus, but also perform unknown biological functions in the cytoplasm.

### 2.4. Construction of Knockout Mutant of Chinese Cabbage Based on CRISPR/Cas9 System

**Figure 5:**
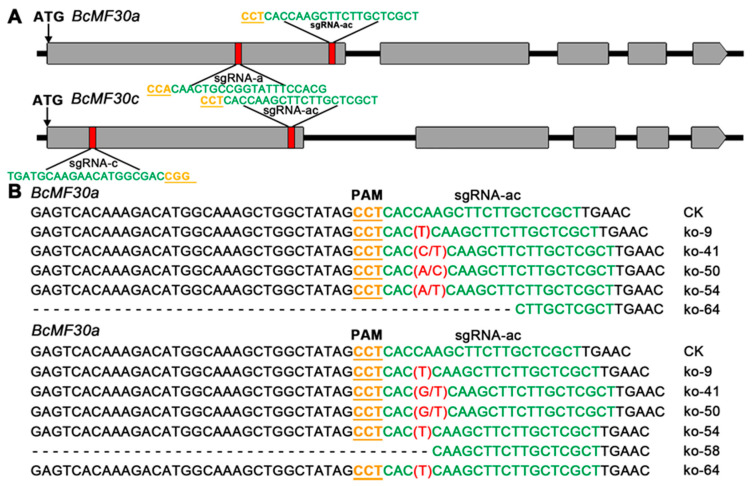
Construction of mutants by using the CRISPR/Cas9 system. (**A**) Genomic location of guide sequences (sgRNA-a, sgRNA-c, and sgRNA-ac) targeting *BcMF30a* and/or *BcMF30c*. (**B**) The gene editing of *BcMF30a* and *BcMF30c* in pCA-sgRNA-ac transgenic T_0_ lines. CRISPR/Cas9: complementation and clustered regularly interspaced short palindromic repeat/CRISPR-associated 9, sgRNA: single-guide RNA, sgRNA-a: sgRNA targeting *BcMF30a*, sgRNA-c: sgRNA targeting *BcMF30c*, sgRNA-ac: sgRNA targeting *BcMF30a* and *BcMF30c*.

In order to explore the biological functions of *BcMF30a* and *BcMF30c*, we planned to construct single-knockout and double-knockout mutants by using the complementation and clustered regularly interspaced short palindromic repeat/CRISPR-associated 9 (CRISPR/Cas9) system. Therefore, we designed a specific single-guide RNA (sgRNA) targeting *BcMF30a* (sgRNA-a) and *BcMF30c* (sgRNA-c) in the first exon, respectively. Meanwhile, we also designed an sgRNA targeting both *BcMF30a* and *BcMF30c* (sgRNA-ac) (Figure 5A). Then, we constructed CRISPR/Cas9 vectors using pBI121 (with *ProAtUBQ*:*Cas9*) and pCAMBIA1301 (with 2 × *CaMV35S*:*Cas9*) as the vector backbones, respectively (both pBI121 and sgRNA-ac contain a *Hind* III cleavage site, so no CRISPR/Cas9 vector containing sgRNA-ac using pBI121 as the backbone has been constructed) (Figure S4). Multiple T_0_ transgenic lines were generated after introducing the constructs into Chinese cabbage calli (Figure S5 and Table S2).

Next, we examined the mutations by sequencing the PCR products amplified from the flanking regions of the target sites in all positive lines. We only detected gene-editing events in 6 of the 31 independent transgenic lines transformed with the pCA-sgRNA-ac vector (Table S2). The sequencing results showed that both *BcMF30a* and *BcMF30c* were edited in the five T_0_ lines (line ko-9, line ko-41, line ko-50, line ko-54, and line ko-64), and in line ko-58, only *BcMF30c* was edited. Further analysis showed that most T_0_ mutants were chimeras with 1-bp insertion (T/C/A in *BcMF30a* and T/G in *BcMF30c*) at exactly 3 bp upstream from the protospacer adjacent motif (PAM) (Figure 5B). The agarose gel electrophoresis results showed that one T_0_ line had a large fragment deletion in *BcMF30a* (line ko-64) and *BcMF30c* (line ko-58), respectively (Figure S6). Unfortunately, lines ko-9 and ko-58 failed to flower in the soil due to poor growth. Moreover, in T_1_ plants, mutations were only detected in the progeny of line ko-41, indicating that the mutations in other lines were not effectively inherited. Results of gene editing analysis showed that most of the T_1_ plants of line ko-41 had biallelic mutations in both *BcMF30a* and *BcMF30c* (Table S3), i.e., insert T or C before the PAM sites of *BcMF30a*, and insert T or G before the PAM sites of *BcMF30c*. Hereafter, these mutants are referred to as *bcmf30a bcmf30c*.

**Figure 6:**
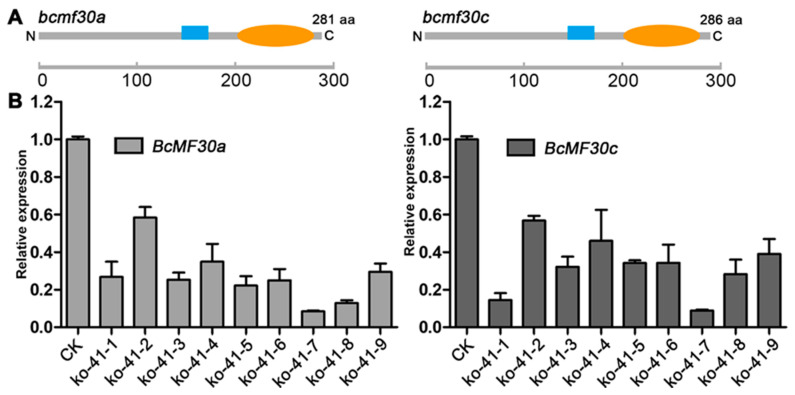
Quantitative detection and protein coding potential prediction of mutated *BcMF30a* and mutated *BcMF30c* in *bcmf30a bcmf30c* mutants. (**A**) Prediction of proteins coded by the mutated *BcMF30a* (*bcmf30a*) and mutated *BcMF30c* (*bcmf30c*). (**B**) The relative expression levels of *bcmf30a* and *bcmf30c* in the inflorescences of *bcmf30a bcmf30c* by qRT-PCR analysis.

Then, we predicted whether the mutated *BcMF30a* and *BcMF30c* still have the potential to encode proteins. The results suggested that the mutated *BcMF30a* and *BcMF30c* may still encode the peptide chains, but their synthesis may be terminated prematurely, and it is predicted that the newly synthesized peptide chains lack the C-terminal of the full-length proteins containing the RRM domain (Figure 6A). Next, we performed qRT-PCR using RNA from *bcmf30a bcmf30c* inflorescence to evaluate whether the mutated *BcMF30a* and *BcMF30c* can be transcribed. The results showed that transcripts can still be detected in *bcmf30a bcmf30c*, but the expression levels of most T_1_ plants were significantly lower than that of the control (Figure 6B).

### 2.5. Mutations in BcMF30a and BcMF30c Do Not Affect Plant Vegetative Growth and Flower Formation

Compared with control plants, the vegetative growth of all T_0_ lines and *bcmf30a bcmf30c* plants did not show any significant differences (Figure S7A,B). Given that *BcMF30a* and *BcMF30c* were specifically expressed in inflorescence, we focused on whether the mutants exhibited defects during reproductive growth. The morphology and size of *bcmf30a bcmf30c* flowers were similar to those of the control plants. Further observation of the sepals, petals, stamens, and pistils also showed no evident abnormality (Figure S7C–P).

### 2.6. bcmf30a bcmf30c Exhibits Partial Male Sterility

**Figure 7:**
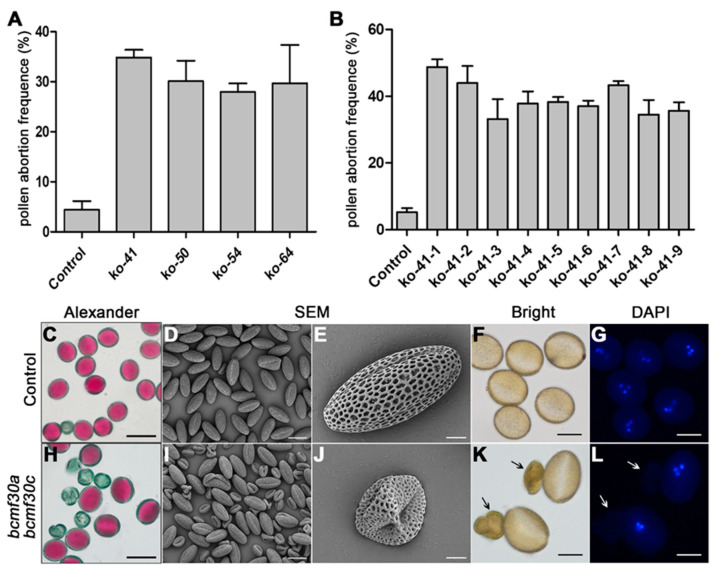
Cytological observation of mature pollen grains in *bcmf30a bcmf30c* mutants of *B. campestris*. Analysis of pollen abortion frequency in control, pCA-sgRNA-ac transgenic T_0_ lines (**A**), and *bcmf30a bcmf30c* mutants (**B**). The values are the mean ± SD. Alexander staining (**C**,**H**), SEM observation (**D**,**E**,**I**,**J**), and 4′,6-diamidino-2-phenylindole (DAPI) staining (**F**,**G**,**K**,**L**) of mature pollen grains in control plants and *bcmf30a bcmf30c* mutants. Bars = 50 μm in (**C**,**H**), 30 μm in (**D**,**I**), 5 μm in (**E**,**J**) and 20 μm in (**F**,**G**,**K**,**L**).

To determine whether mutations in *BcMF30a* and *BcMF30c* cause abnormal pollen development, we conducted pollen viability testing and pollen morphology observation. The results of Alexander staining showed that on average, about 30.7% of pollen grains from T_0_ lines were aborted as compared with only 4.4% from the control plants (Figure 7A). Further analysis showed that up to 39.2% of *bcmf30a bcmf30c* mature pollen grains were nonviable (Figure 7B,C,H). Scanning electron microscopy (SEM) observation also demonstrated that partial mature pollen from *bcmf30a bcmf30c* were shrunken and collapsed, but the other part of the mature pollen grains had no significant difference compared with the control pollen grains in morphology and size (Figure 7D,E,I,J). 4′,6-diamidino-2-phenylindole (DAPI) staining showed that no nuclei were observed in aberrant pollen from *bcmf30a bcmf30c*, whereas normal-shaped pollen grains contain three nuclei (Figure 7F,G,K,L).

**Figure 8:**
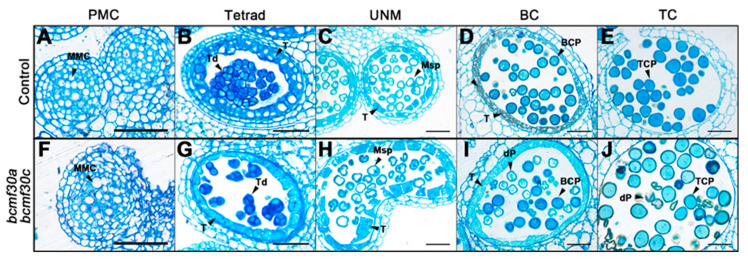
The semi-thin transverse sections of *bcmf30a bcmf30c* anthers. Semi-thin sections of anthers from the control plants (**A**–**E**) and *bcmf30a bcmf30c* (**F**–**J**) at the pollen mother cell stage (PMC, (**A**,**F**)), tetrad stage (Tetrad, (**B**,**G**)), uninucleate microspore stage (UNM, (**C**,**H**)), bicellular pollen stage (BC, (**D**,**I**)) and tricellular pollen stage (TC, (**E**,**J**)). BCP, bicellular pollen; MMC, microspore mother cell; Msp, microspore; P, pollen; dP, degenerated pollen; T, tapetum; TCP, tricellular pollen; Tds, tetrads. Bars = 25 μm.

In order to clarify the anther development process in *bcmf30a bcmf30c* and to investigate the precise stage when pollen began to develop abnormally due to the mutations, semi-thin transverse sections were performed. No detectable differences between *bcmf30a bcmf30c* and the control pollen were observed inside the anther locules before the uninucleate stage (Figure 8A–C,F–H). Visible abnormalities appeared in the anthers of *bcmf30a bcmf30c* at the bicellular stage, in which a large number of pollen grains were deformed and stained less (Figure 8D,I). By the tricellular stage, the pollen inclusion was almost completely degraded (Figure 8E,J).

**Figure 9:**
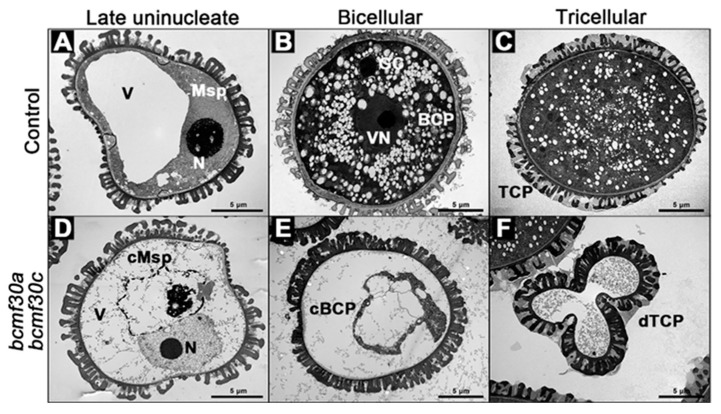
Transmission electron micrographs of pollen grains from *bcmf30a bcmf30c* mutants. Ultrastructure of pollen at different developmental stages from the control plants (**A**–**C**) and *bcmf30a bcmf30c* (**D**–**G**). (**A**,**D**) Late uninucleate stage; (**B**,**E**) bicellular pollen stage; (**C**,**F**,**G**) tricellular pollen stage. BCP, bicellular pollen; cBCP, collapsed BCP; GC, generative cell; Msp, microspore; cMsp, collapsed Msp; N, nucleus; TCP, tricellular pollen; cTCP, collapsed TCP; dTCP, degenerated TCP; V, Vacule; VN, vegetative nucleus. Bars = 5 μm.

The abnormalities of the *bcmf30a bcmf30c* pollen grains were further determined by transmission electron microscopy (TEM). As shown in Figure 9, the nucleus in the control microspore was displaced to the side by a large vacuole at the late uninucleate stage (Figure 9A). However, during this stage, although the *bcmf30a bcmf30c* microspores contain normally developed nuclei, most of the cytoplasm has been degraded (Figure 9D). By the bicellular stage, no nucleus was observed in *bcmf30a bcmf30c* pollen (Figure 9E). At the tricellular stage, mature control pollen grains were trinuclear with dense cytoplasm (Figure 9C). However, the degradation of microspores in *bcmf30a bcmf30c* plants resulted in aborted pollen that lacked any cytoplasmic components, leaving only the pollen wall that appeared to be intact (Figure 9F).

### 2.7. Pollen Germination Were Significantly Affected in bcmf30a bcmf30c

**Figure 10:**
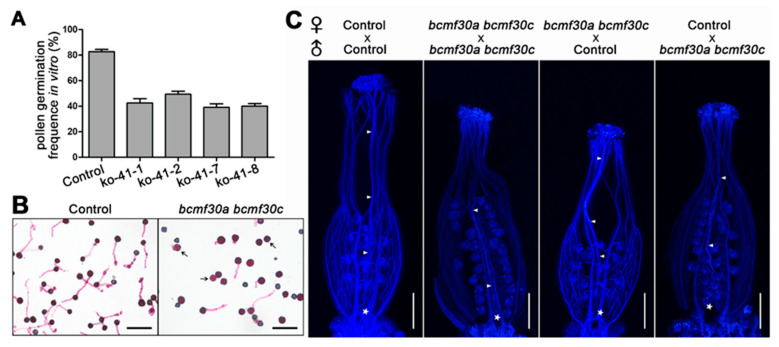
The in vitro and in vivo germination tests of *bcmf30a bcmf30c* mature pollen. (**A**) Pollen germination frequencies of *bcmf30a bcmf30c* pollen in vitro. Only viable pollen was counted for pollen germination rate statistics. The values are the mean ± SD. (**B**) Control and *bcmf30a bcmf30c* mature pollen germinated in vitro for 4 h. (**C**) Aniline blue staining of pollen tubes in pistils after self-pollination and cross-pollination for 12 h. The arrows indicated the pollen tubes and the asterisk indicated the end position of pollen tubes. Bars = 100 μm.

To evaluate the effects of mutations in *BcMF30a* and *BcMF30c* on pollen germination, in vitro and in vivo pollen germination tests were conducted. In both control plants and *bcmf30a bcmf30c* plants, only viable pollen (identified by Alexander staining and morphological observation) was counted for pollen germination rate statistics. In vitro, less than half (39.2–49.4%) of viable pollen grains from *bcmf30a bcmf30c* plants geminate normally, which is significantly lower than the germination rate in the control plants (> 80%) (Figure 10A,B). The difference of pollen tube growth in pistils between the *bcmf30a bcmf30c* plants and the control plants was also observed. The results revealed that the number of *bcmf30a bcmf30c* pollen tubes was much less than that of control pollen tubes, whether they were grown in the pistils of *bcmf30a bcmf30c* plants or the control plants (Figure 10C). These results demonstrated that mutations in *BcMF30a* and *BcMF30c* not only affect pollen development, but also pollen germination.

## 3. Discussion

Many species of Brassicaces are cultivated worldwide as oil seed or vegetable crops and display strong hybrid vigor or heterosis. Thus, hybrid breeding has become one of the main strategies for ensuring the production of Brassicaceae crops, including Chinese cabbage, which is one of the most important leafy vegetables in Asia. The identification of male fertility-related genes and the elucidation of the genetic, molecular, and biochemical mechanisms of male sterility caused by the functional loss of these genes are the top priority of both basic research and application of Chinese cabbage breeding. In this study, we identified 11 male fertility-related CCCH zinc-finger protein genes in Chinese cabbage and characterized the roles of two homologues, *BcMF30a* and *BcMF30c*, in pollen development and pollen germination for the first time. This work further enriched the functional studies of CCCH genes in plants, especially in male fertility.

### 3.1. Male Fertility-Related CCCH Zinc-Finger Protein Genes in B. campestris

As an important process during reproductive development, a large number of genes are expressed during pollen development, including the CCCH zinc-finger protein genes [45]. Recently, functional studies on some plant CCCH genes have been conducted, which demonstrated that these genes can act as vital regulators during both plant development and stress response, but much remains to be revealed. So far, only a handful of genes have been shown to be involved in pollen development. Moreover, these genes are mainly from model plants, including *AtC3H14*, *AtC3H15*/*CDM1* in Arabidopsis, and *DCM1* in rice [20,21,26]. In this study, we identified 11 male fertility-related CCCH zinc-finger protein genes in *B. campestris*. Surprisingly, among these 11 genes, three of them (Bra004045, *BcMF30a*, and *BcMF30c*) were identified as CCCH zinc-finger protein genes for the first time, which were not included in the 63 genes identified by Rameneni et al. (2018) [12] and the 103 genes identified by Pi et al. (2018) [11] as the CCCH zinc-finger protein genes in *Brassica rapa*. This result implies that the CCCH zinc-finger protein gene family in *B. campestris* contains a large number of members, and some members are likely to be missed when predicted in silico.

The expression profiles of some CCCH genes indicated that they may be involved in various abiotic stress responses, such as cold, drought, and salt stress responses [11,12]. To our knowledge, this is the first report linking the CCCH genes of Chinese cabbage to pollen development, and none of these 11 genes has been functionally studied. Among them, only one orthologous gene in Arabidopsis, *AtC3H15*/*CDM1* (the homologous gene of Bra004288), has been revealed to be required for male fertility by regulating callose metabolism during microsporogenesis [20]. Here, we also found that Bra004288 was specifically expressed at the tetrad stage, which is a period when the newly formed microspores were blanketed and separated by a thick callose wall. Notably, there are three other CCCH genes (Bra030149, Bra030151, and Bra032326) specifically expressed by the tetrad (Figure 1A), suggesting that they may also be involved in the formation of tetrads. Several other genes exhibit different spatiotemporal expression patterns (Figure 1A), indicating that they may function at different pollen developmental stages. In this study, functional studies of *BcMF30a* and *BcMF30c*, which began to be expressed at the uninucleate stage (Figure 2), confirmed that they are required for microgametogenesis. The regulatory functions of these male fertility-related CCCH genes in pollen development need further research.

### 3.2. BcMF30a and BcMF30c, Two Novel Non-TZF Proteins, May Play a Dual Role in Plant Cells

Based on the selected male fertility-related CCCH zinc-finger protein genes, we further isolated and characterized a pair of paralogous genes from Chinese cabbage flower buds, named *BcMF30a* and *BcMF30c*, which both encode novel non-TZF proteins with only one CCCH motif. As mentioned above, although two research studies have predicted the members of CCCH zinc-finger protein gene family in *Brassica rapa* [11,12], this study confirmed for the first time that *BcMF30a* and *BcMF30c* are also members of this family.

Similar to other types of zinc finger proteins that usually act as transcription factors, recent studies have shown that some CCCH zinc-finger proteins can bind to DNA and regulate gene expression at the transcriptional level. For example, the human CCCH zinc-finger protein, mini-chromosome maintenance 10 (Mcm10), is one of the DNA-replication factors that can serve as a physical link between the DNA polymerases and Mcm2-7 complex, and its central domain containing the CCCH motif can bind to DNA and several proteins [46]. Fetal liver zinc finger protein 1 (Fliz1), a zebrafish CCCH zinc-finger protein, acts as a transcriptional repressor that can specifically bind to the negative *cis*-acting element of *GATA-3* [47]. In contrast, AtC3H17 in Arabidopsis and IbC3H18 in sweet potato both function as nuclear transcriptional activators [37,38]. Rice Leaf and tiller angle Increased Controller (OsLIC) and Arabidopsis AtC3H14 and AtC3H15/CDM1 can also bind to DNA and show transcriptional activity in yeast [21,48]. Here, we also found that BcMF30a and BcMF30c can exhibit transcriptional activation activity in yeast (Figure 3). Subcellular localization analysis showed that they can be localized to the nucleus, although the signals of both BcMF30a/BcMF30c-eGFP and eGFP-BcMF30a/BcMF30c fusion proteins in the nucleus were weaker than those of free-eGFP (Figure 4 and Figure S3). These findings demonstrated that BcMF30a and BcMF30c may function as nuclear transcriptional activators in plant cells. *IbC3H18* is the most likely homologous gene of *BcMF30a* and *BcMF30c* in sweet potato. The study showed that the 303 C-terminal amino acid residues (containing an RRM domain) of IbC3H18 was responsible for its transcriptional activation activity [41]. Therefore, we speculate that the transcriptional activation domains of BcMF30a and BcMF30c probably are located at the C-terminus of the proteins, which requires further research in the future.

Mounting research studies have shown that many identified CCCH zinc-finger proteins can also function as RNA-binding proteins and regulate gene expression at the post-transcriptional level. For instance, tristetraprolin (TTP), a mouse CCCH zinc-finger protein, can regulate mRNA turnover by specifically binding to the AU-rich elements in the 3’ untranslated regions of target mRNAs [49]. In Arabidopsis, HUA1 and Cleavage and Polyadenylation Specificity Factor 30 (AtCPSF30) are both RNA-binding proteins that can facilitate the maturation of the *AGAMOUS* pre-mRNA and regulate calmodulin-mediated RNA processing, respectively [36,50]. Several TZF proteins, such as AtTZF1, AtTZF9, AtC3H14, and AtC3H15/CDM1 in Arabidopsis, and OsTZF1 in rice, also show RNA-binding activity [13,21,23,30]. Unlike typical transcription factors that are usually localized only in the nucleus, in this study, we found that BcMF30a and BcMF30c have obvious signals in the cytoplasm in addition to the nucleus (Figure 4 and Figure S3), which suggests that they also play a role in the cytoplasm. In addition to containing a CCCH motif, BcMF30a and BcMF30c both contain a LOTUS domain and an RRM domain, both of which are putative RNA-binding domains [51,52,53], implying that they are likely to have RNA-binding activity. In fact, multiple CCCH zinc-finger proteins, such as AtTZF1, AtC3H14, and AtC3H15/CDM1 have been found to have a dual nuclear and cytoplasmic localization and can bind to DNA as well as RNA in vitro [13,21]. These results indicate that BcMF30a and BcMF30c may function as RNA-binding proteins in the cytoplasm.

Together, we believe that BcMF30a and BcMF30c are two novel non-TZF proteins in *B. campestris*, and they may play a dual role in plant cells, i.e., act as transcriptional activators in the nucleus and function as RNA-binding proteins in the cytoplasm. The next critical work toward understanding the molecular role of BcMF30a and BcMF30c in plant cells would be the identification of target genes transcriptionally activated by them and target RNAs that bind them.

### 3.3. BcMF30a and BcMF30c Are Required for Microgametogenesis and Pollen Germination

In flowering plants, the pollen grains produced during the reproductive growth stage harbor haploid male germ cells, which are vital for sexual reproduction. Pollen development takes place in the anther locules and can be divided into two major phases: microsporogenesis and microgametogenesis [54]. During microsporogenesis, the primary sporogenous cells produce the microsporocytes and then undergo meiosis to give rise to tetrads of haploid microspores enclosed and separated by thick callosal walls, which are subsequently released as individual microspores by callose enzyme [55]. During microgametogenesis, microspores successively undergo substantial cell growth, asymmetric division at pollen mitosis I (PMI), and germ cell division at PMII to form tricellular pollen, in which two sperm cells are engulfed by the vegetative cell [56]. Our expression analysis data showed that during pollen development, *BcMF30a* and *BcMF30c* began to express in pollen at the microspore stage, peaked at the bicellular stage, and continued to express in mature pollen (Figure 1A and Figure 2). Then, we investigated whether pollen development in the *bcmf30a bcmf30c* mutant was affected. Our analyses showed that partial pollen were aborted in the mature pollen stage (Figure 7). Semi-thin sections and TEM results demonstrated that the *bcmf30a bcmf30c* pollen developed normally during the microsporogenesis phase. However, the cytoplasm of microspores started to degrade at the late microspore stage, and it disappeared completely at the tricellular pollen stage, leaving only the pollen exine (Figure 8 and Figure 9). Therefore, it is likely that the *bcmf30a bcmf30c* mutation did not affect the formation of microspores, but it did disrupt the microgametogenesis.

To date, it is expected that near 100 genes affecting pollen development and cellular functions have been isolated in *Arabidopsis* by several genetic screening strategies [54,57]. The phenotypic analyses of certain gametophytic mutants indicate that some genes are specifically involved in cell division and patterning. For example, *DUO POLLEN1* (*DUO1*) and its direct targets, *DUO1*-*ACTIVATED ZINC FINGER PROTEIN1* (*DAZ1*) and *DAZ2*, form a *DUO1*-*DAZ1*/*2* network model to control G2- to M-phase transition and gamete differentiation [58,59]. However, mutations in most of other genes often lead to pollen degeneration and abortion at various developmental stages. For instance, mutations in Arabidopsis *ABNORMAL GAMETOPHYTE* (*AGM*), *LONG-CHANIN BASE1* (*LCB1*)/*LCB2A*/*LCB2C*, and cellulose synthase genes (*CESA1*/*2*/*3*/*6*/*9*) can cause pollen abnormality at the microspore stage, bicellular stage, and tricellular stage, respectively [60,61,62]. Overall, proteins with a variety of functions are involved in various processes of pollen development, including chromatin organization, cell division and differentiation, cell wall construction, energy, metabolism, ion transport, and transcriptional regulation. As mentioned above, in view that both BcMF30a and BcMF30c may play a dual role in plant cells, we believe that they are involved in the microgametogenesis at both transcriptional and post-transcriptional levels. As for which biological processes are specifically affected, further research is urgently needed to clarify.

Pollen germination is also strictly regulated, which is a prerequisite for successful fertilization. At present, many genes essential for pollen germination have been identified, most of which are positive regulators, including *NO POLLEN GERMINATION1* (*NPG1*) and *NPG1-related* genes, which encode Ca^2+^ sensor calmodulin (CaM)-binding proteins [63]. However, there are also a few negative regulators, such as *JINGUBANG* (*JGB*) and *GTP-BINDING PROTEIN RELATED1* (*GPR1*), which can maintain the optimal pollen germination speed [64,65]. Here, we also observed the promoter activity of *BcMF30a* and *BcMF30c* in germinated pollen and pollen tubes (Figure 2E,F). Moreover, many viable *bcmf30a bcmf30c* pollen grains cannot germinate normally both in vitro and in vivo (Figure 10). These findings suggest that *BcMF30a* and *BcMF30c* are also required for pollen germination and probably act as positive regulators. Notably, although this study only characterized the phenotype of *bcmf30a bcmf30c* double mutants, given that *BcMF30a* and *BcMF30c* share high amino acid identity and have similar spatiotemporal expression patterns, we believe they are functional redundant.

Although the characterization of the *bcmf30a bcmf30c* mutants provides insights into the role of the pollen-specific *BcMF30a* and *BcMF30c* genes, the molecular mechanisms of how they regulate pollen development and pollen germination and whether and how they cooperate to play roles of transcription factors and RNA-binding proteins to achieve transcriptional and post-transcriptional regulation remain largely unknown. Investigation of these issues will provide further insights into the molecular basis regulating male gametophyte development.

## 4. Materials and Methods

### 4.1. Plant Material and Growth Conditions

The *B. campestris* GMS A/B line system ‘*Bcajh97-01A/B*’ was planted in the experimental farm of Zhejiang University, Hangzhou, China. *Brassica campestris* L. ssp. *chinensis* var. *parachinensis* cv. Youqing 49 was used for the genetic transformation experiments due to its precocity characteristic. The transgenic Chinese cabbage of T_0_ lines, T_1_ plants, transgenic *Arabidopsis thaliana* Columbia-0 plants, and H2B-RFP transgenic tobacco (*Nicotiana benthamiana*) plants were cultivated in a phytotron under long-day conditions (22 °C, 16 h light/18 °C, 8 h dark).

### 4.2. Expression Analysis of Male Fertility-Related CCCH Zinc-Finger Protein Genes in B. campestris

In our previous study, we have obtained RNA-Seq data of ‘*Bcajh97-01A/B*’ at five typical flower bud development stages [44]. Since the only difference between the fertile line ‘*Bcajh97-01B*’ and sterile line ‘*Bcajh97-01A*’ is that ‘*Bcajh97-01A*’ is a male meiotic cytokinesis mutant that cannot produce any viable mature pollen grains, it is an ideal strategy to identify the CCCH zinc-finger protein genes that may be involved in male fertility by comparing the expression levels of these genes in ‘*Bcajh97-01A/B*’. The data shown in Table S1 were collected from the TAIR (http://www.arabidopsis.org/) and Brassica (http://brassicadb.org/brad/) databases. Protein structure prediction was performed using SMART (http://smart.embl-heidelberg.de/).

### 4.3. RT-PCR and qRT-PCR

Total RNAs were extracted by using RNAiso Plus (Takara, Shiga, Japan). Then, RNAs were transcribed into cDNAs with a PrimerScript RT reagent Kit (Takara, Shiga, Japan) and used as templates for RT-PCR and qRT-PCR analysis with specific primers (Table S4). *UBC10* was used as the internal control. qRT-PCR was conducted by using a SYBR^®^ Premix Ex Taq™ Kit (Takara, Japan) on a CFX96 Real-Time System (Bio-Rad, Hercules, CA, USA). Results were calculated using the 2^−ΔΔCt^ method. Three technical repeats were performed.

### 4.4. β-Glucuronidase (GUS) Histochemical Staining Assay

The promoter sequences upstream of the ATG codons of *BcMF30a* (1720-bp) and *BcMF30c* (1919-bp) were amplified with gene-specific primers (Table S4) and cloned into pBI101 vector flanking the *GUS* reporter gene to create the fusion constructs, *ProBcMF30a:GUS* and *ProBcMF30c:GUS*. The floral-dip method mediated by *Agrobacterium* was used to transfer plasmids into *Arabidopsis* plants [66]. Transgenic plants were screened in MS agar plates containing kanamycin. GUS staining of inflorescences and germinated pollens were performed as described by Mudunkothge et al. (2014) [67].

### 4.5. Transcriptional Activation Assay in Yeast

The full lengths of *BcMF30a* and *BcMF30c* coding sequences were obtained by PCR with specific primers and cloned into the *pGBK-T7* vector. Then, the *pBD-BcMF30a*, *pBD-BcMF30c*, *pBD-AD* (positive control), and *pBD-GAL4* (negative control) plasmids were transfected into the yeast strain (AH109). The transformed yeast cells were grown on SD/-Trp and SD/-Trp/-His/-Ade plates (lacking threonine, histidine, and adenine) at 30 °C for 3 days. The transcriptional activation activities of *BcMF30a* and *BcMF30c* were evaluated according the growth status of yeast cells on screening plates. The primers used are listed in Table S4.

### 4.6. Subcellular Localization

To observe the subcellular localization of *BcMF30a* and *BcMF30c*, we amplified the coding sequences with specific primers (Table S4) and then inserted into the *pFGC-eGFP* vector to form *pFGC-BcMF30a/c-eGFP* and *pFGC-eGFP-BcMF30a/c*. Then, the fusion vectors were transiently transformed into 4-week-old H2B-RFP transgenic tobacco leaves by an infiltrated method. Fluorescent signals were analyzed 2 days after transformation under a laser confocal scanning microscope (Nikon, A1, Tokyo, Japan).

### 4.7. Construction of CRISPR/Cas9 Vectors and Generation of Mutants

For each *BcMF30a* and *BcMF30c* targeting site, two complementary oligonucleotides with 20-bp target sequences (sgRNA-a, sgRNA-c, and sgRNA-ac) were synthesized (Table S4). Each set of oligo pairs was annealed to generate double-stranded DNA with 4-bp overhangs on both ends, which were cloned into the *Bbs* I site of vectors containing the *pAtU6-26:sgRNA-scaffold*. According to the procedures shown in Figure S4, the CRISPR/Cas9 vectors targeting *BcMF30a* (pBI-sgRNA-a, pCA-sgRNA-a), *BcMF30c* (pBI-sgRNA-c, pCA-sgRNA-c), and targeting both *BcMF30a* and *BcMF30c* (pCA-sgRNA-ac) were constructed. Then, all CRISPR/Cas9 vectors were transformed into the fertile Chinese cabbage (Youqing 49) by an *Agrobacterium*-mediated transformation method as described previously [68]. The DNA of generated T_0_ lines was extracted and then analyzed by a PCR screening approach with specific primers. For all the positive transgenic lines, the PCR products amplified from the flanking regions of the target sites were sequenced to determine mutations in *BcMF30a* and *BcMF30c*. For T_0_ line plants with mutations, the T_1_ plants were propagated. Then, gene mutations in T_1_ plants were also confirmed by PCR and sequencing. The expression of mutated *BcMF30a* and *BcMF30c* were analyzed by qRT-PCR according to the method mentioned above. The primers used are listed in Table S4.

### 4.8. Phenotypic and Cytological Observation of Pollen and Pollen Germination Assays

When performing phenotypic and cytological observations, tissue-cultured plants without transgenic events were used as controls for T_0_ lines, and plants grown from seeds were used as controls for *bcmf30a bcmf30c* plants. The mature pollen grains were dyed with Alexander stain [69] to investigate the pollen viability. DAPI solution [70] was used to detect the nuclei in pollen grains. The stained pollen grains were observed and photographed under a fluorescent microscope (Nikon, ECLIPSE 90i, Tokyo, Japan). For SEM, pollen grains were smeared on SEM carriers, and then the sample was coated with gold-palladium in Hitachi MC1000 ion sputter for about 10 min. The images were observed and captured with a microscope (GeminiSEM 300, Carl Zeiss Microscopy GmbH, Oberkochen, Germany). TEM analyses as well as in vitro and in vivo pollen germination tests were conducted as described by Lin et al. (2014) [71] with some modifications. The samples for TEM observation were also used for semi-thin transverse sections observation. The detailed procedures referred to Lin et al. (2018) [72].

## 5. Conclusions

Collectively, we identified 11 male fertility-related CCCH zinc-finger protein genes in *B. campestris* for the first time. Moreover, we further isolated and characterized two non-TZF genes, *BcMF30a* and *BcMF30c*, which were specifically expressed in pollen during microgametogenesis and germinated pollen and pollen tubes. Molecular function analysis indicated that both BcMF30a and BcMF30c may play a dual role as transcription factors and RNA-binding proteins in plant cells. Knockout of *BcMF30a* and *BcMF30c* by using CRISPR/Cas9 technology results in partial pollen abortion and a significant reduction in pollen germination. These results indicate that *BcMF30a* and *BcMF30c* play important roles in the pollen development and pollen germination of Chinese cabbage, and they are likely to function at both the transcriptional and post-transcriptional regulatory pathways.

## Supplementary Materials

Supplementary Materials can be found at https://www.mdpi.com/1422-0067/21/17/6428/s1. Table S1. Detailed information of male fertility-related CCCH zinc-finger genes in *Brassica campestris* and information relevant to *Arabidopsis thaliana*. Table S2. T_0_ lines and gene editing of plants transformed by CRISPR-Cas9 vectors. Table S3. Genotypes of 9 *bcmf30a bcmf30c* plants generated by line ko-41. Table S4. Sequence of primers used in this study. Figure S1. The exon-intron configurations of 11 male fertility-related CCCH zinc-finger protein genes in *Brassica campestris*. Figure S2. Sequence alignment of BcMF30a and BcMF30c. Figure S3. Subcellular localization of BcMF30a-eGFP and BcMF30c-eGFP fusion proteins in tobacco epidermal cells. Figure S4. Flow chart of constructing CRISPR-Cas9 knockout vectors. Figure S5. PCR analyses of transgenic T_0_ lines transformed with CRISPR/Cas9 knockout vectors of *BcMF30a* and *BcMF30c*. Figure S6. PCR analyses of *BcMF30a* and *BcMF30c* in pCA-sgRNA-ac transgenic T_0_ lines. Figure S7. Morphological observation of plants and floral organs of *bcmf30a bcmf30c* plants.

## Abbreviations

ABA	abscisic acid
bp	base pair
GA	gibberellin
CaMV	cauliflower mosaic virus
CCCH	Cysteine-cysteine-cysteine-histidine
CRISPR/Cas9	complementation and clustered regularly interspaced short palindromic repeat/CRISPR-associated 9
GFP	green fluorescent protein
GMS	genetic male sterile
GUS	β-glucuronidase
LOTUS	Limkain, Oskar, and TUdor-containing proteins 5 and 7
PAM	protospacer adjacent motif
PCR	polymerase chain reaction
PMI	pollen mitosis I
PMII	pollen mitosis II
Pre-mRNA	precursor messenger RNA
qRT-PCR	quantitative reverse transcriptase polymerase chain reaction
RNA-seq	RNA sequencing
RRM	RNA recognition motif
SEM	scanning electron microscopy
TEM	transmission electron microscopy
TZF	tandem CCCH zinc-finger
