# Supplementary material for: BcMF30a and BcMF30c, Two Novel Non-Tandem CCCH Zinc-Finger Proteins, Function in Pollen Development and Pollen Germination in Brassica campestris ssp. chinensis
Source: Int J Mol Sci. 2020 Sep 3;21(17):6428. doi: 10.3390/ijms21176428 (PMC7504113; doi:10.3390/ijms21176428)
Supplement: Supplementary file 1 [file ijms-21-06428-s001.zip › Supplementary Figures and Tables/Figure S1-S7.pdf]

International Journal of
Molecular Sciences

MDPI

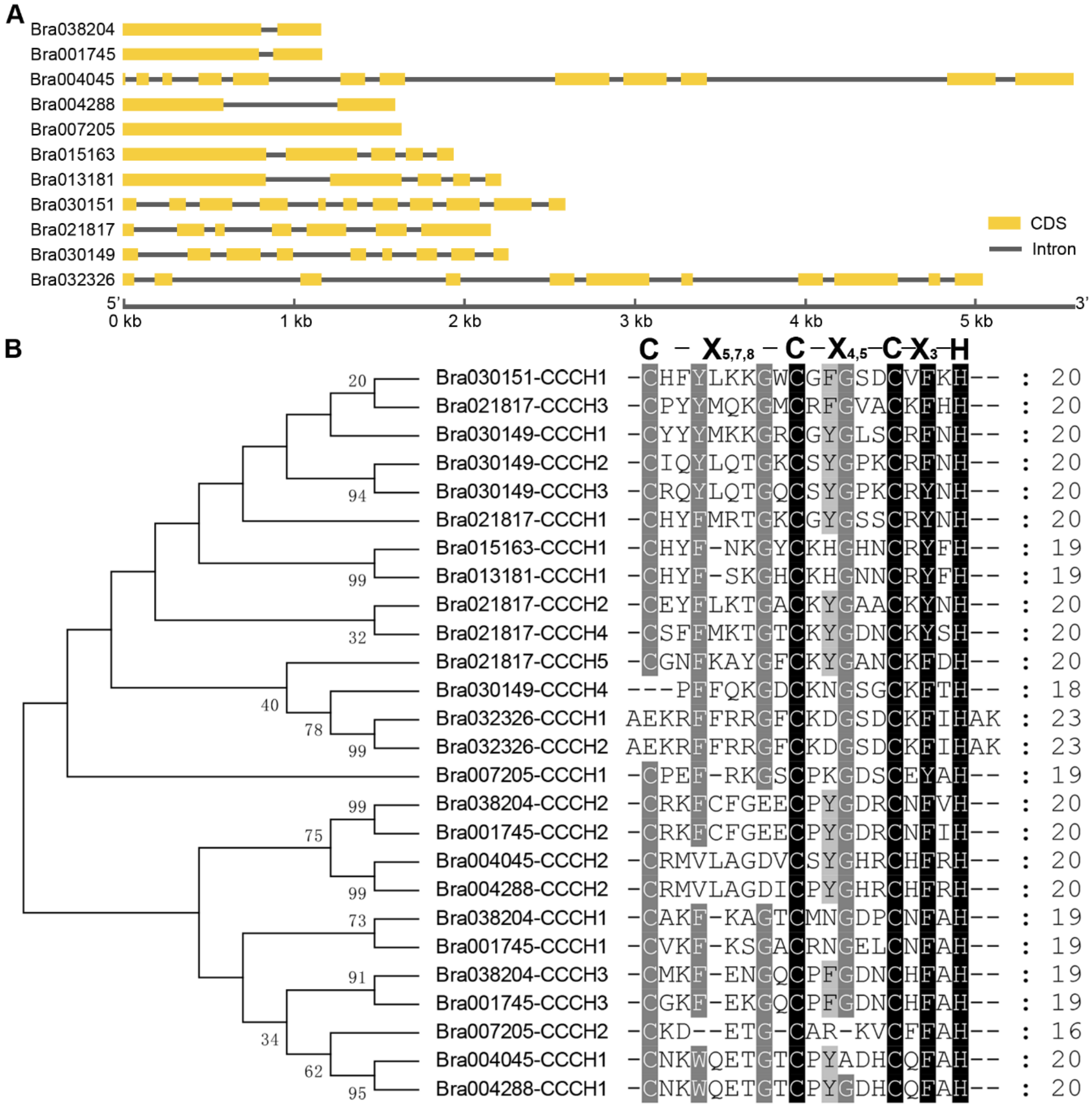

Figure S1. The exon-intron configurations of 11 male fertility-related CCCH zinc-finger protein genes in *B. campestris* (**A**). Phylogenetic relationships and multiple sequence alignment of CCCH zinc finger motifs from 11 male fertility-related CCCH zinc-finger proteins (**B**).

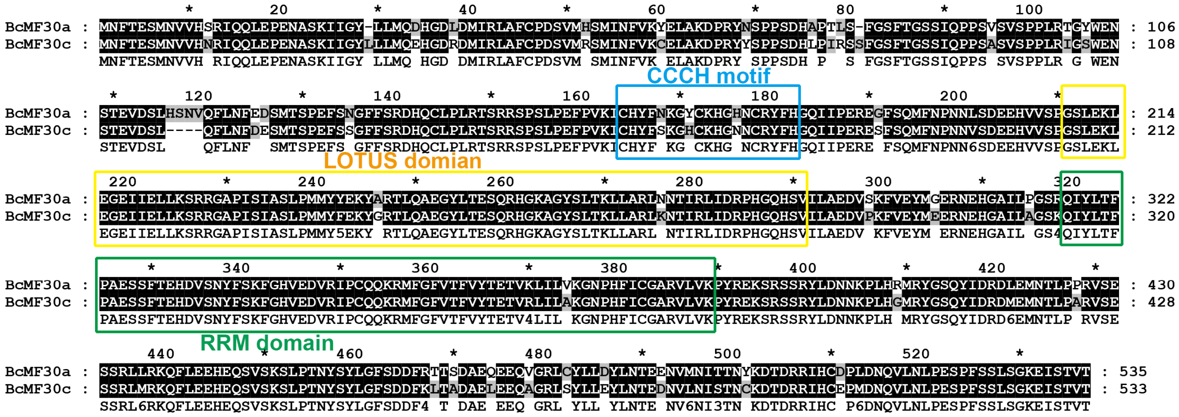

Figure S2. Sequence alignment of BcMF30a and BcMF30c. Both BcMF30a and BcMF30c contain a CCCH motif, a LOTUS domain and an RRM domain.

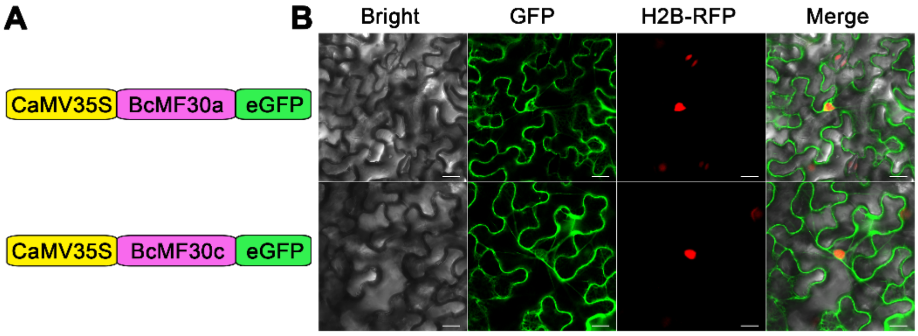

Figure S3. Subcellular localization of BcMF30a-eGFP and BcMF30c-eGFP fusion proteins in tobacco epidermal cells. (**A**) Constructs used for the subcellular localization analysis. (**B**) Both BcMF30a-eGFP and BcMF30c-eGFP were located in nucleus and dispersed in the cytoplasm of tobacco epidermal cells. H2B is a marker protein of nucleus. Pictures represent white field images (Bright), epifluorescence (GFP and RFP) and merged images (Merge). Bar =25 μm.

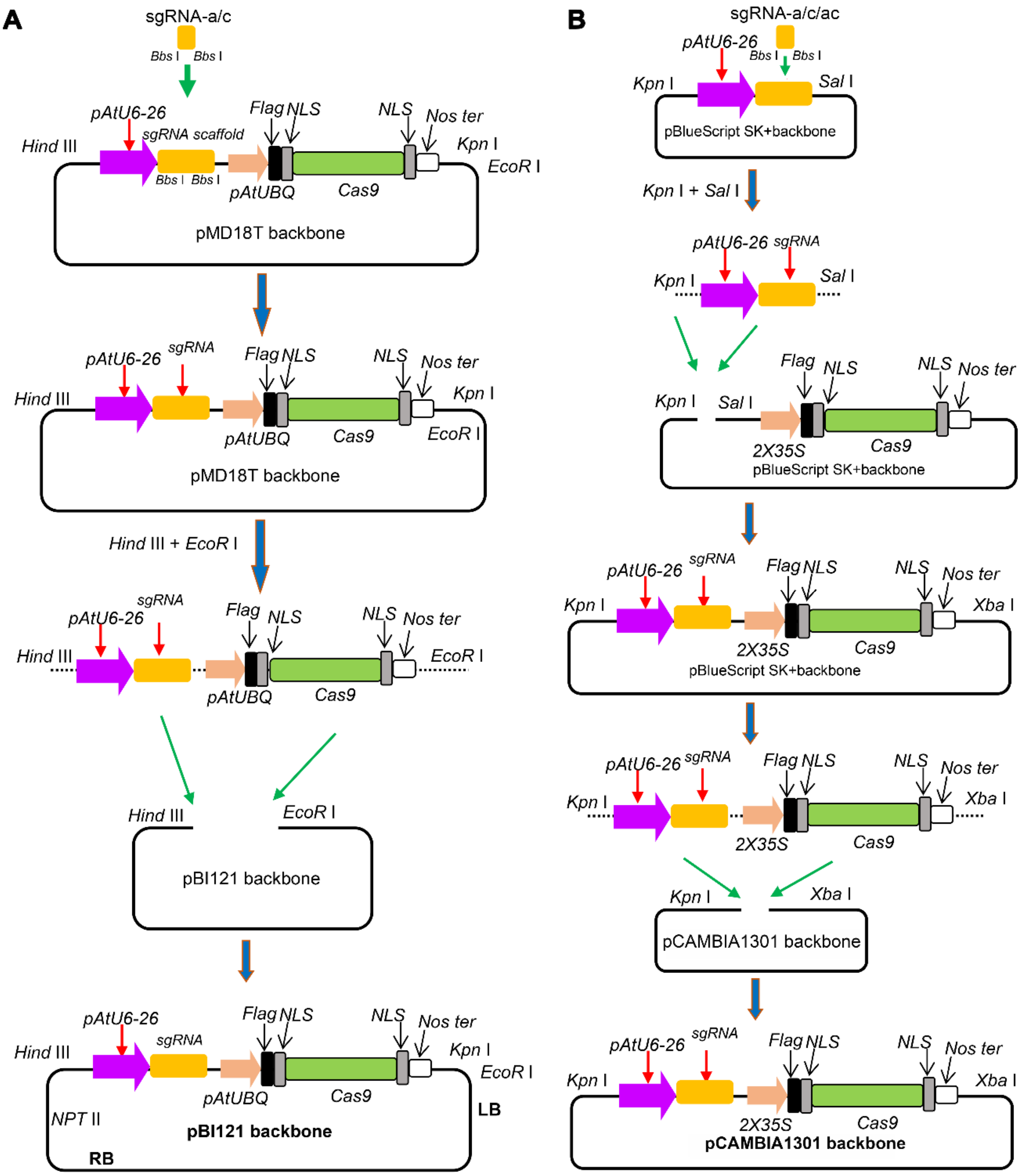

Figure S4. Flow chart of constructing CRISPR-Cas9 knockout vectors. (**A**) pBI121 as vector backbone, and *Cas9* gene is activated by promoter of *AtUBQ*. (**B**) pCAMBIA1301 as vector backbone, and *Cas9* gene is activated by 2× *CaMV35S*.

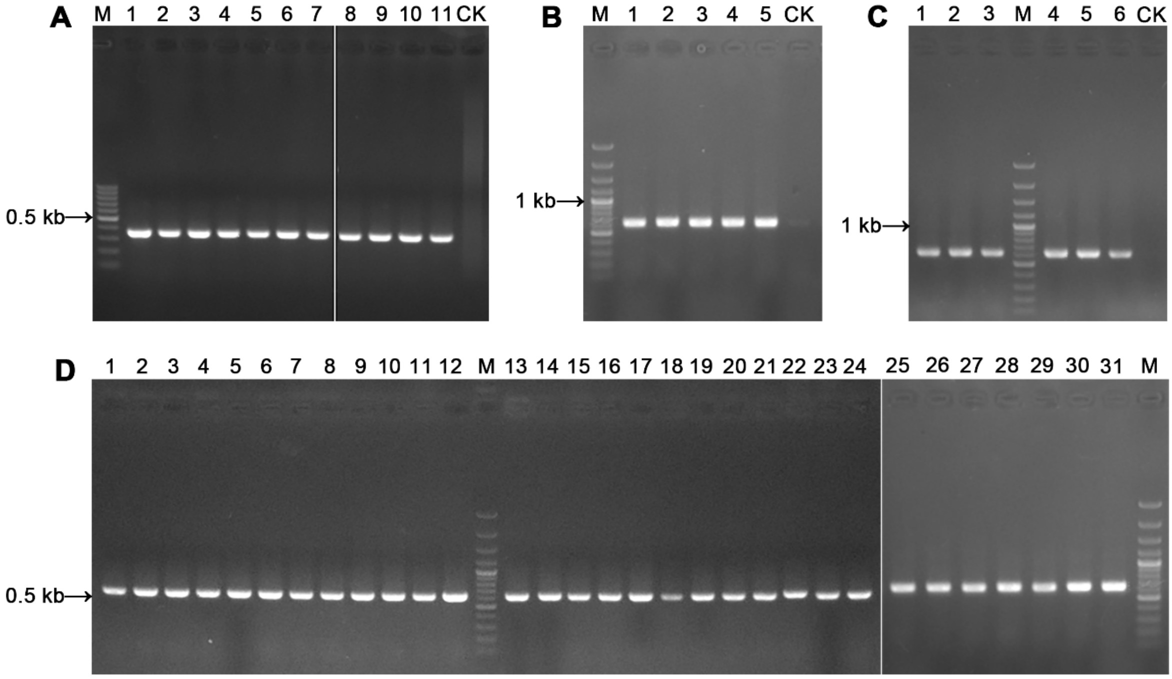

Figure S5. PCR analyses of transgenic T_0 lines transformed with CRISPR/Cas9 knockout vectors of *BcMF30a* and *BcMF30c*. Transgenic plants transformed with pBI-sgRNA-a (**A**, lanes 1 to 7), pBI-sgRNA-c (**A**, lanes 8 to 11), pCA-sgRNA-a (**B**, lanes 1 to 5), pCA-sgRNA-c (**C**, lanes 1 to 6), pCA-sgRNA-ac (**D**, lanes 1 to 31). Lanes CK indicated the negative control. Lanes M indicated DNA molecular marker.

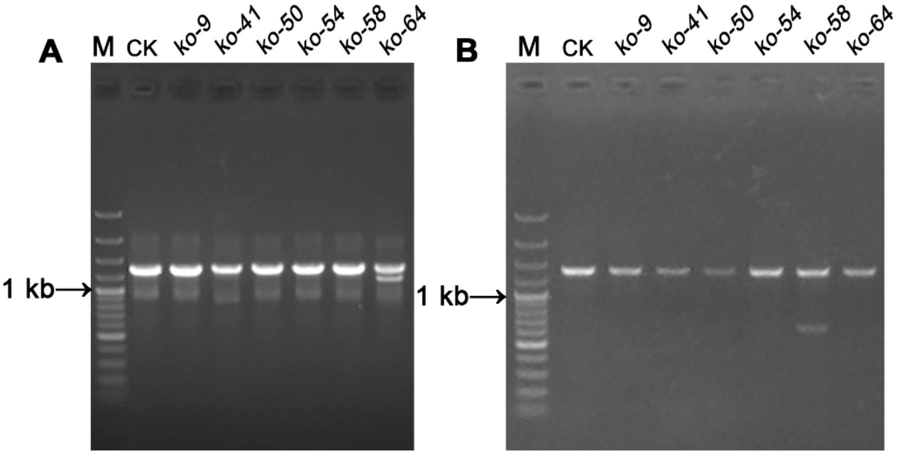

Figure S6. PCR analyses of *BcMF30a* and *BcMF30c* in pCA-sgRNA-ac transgenic T_0 lines. (**A**) The amplification results of *BcMF30a*. About 200-bp deletion in *BcMF30a* was occurred in ko-64. (**B**) The amplification results of *BcMF30c*. About 850-bp deletion in *BcMF30c* was occurred in ko-58.

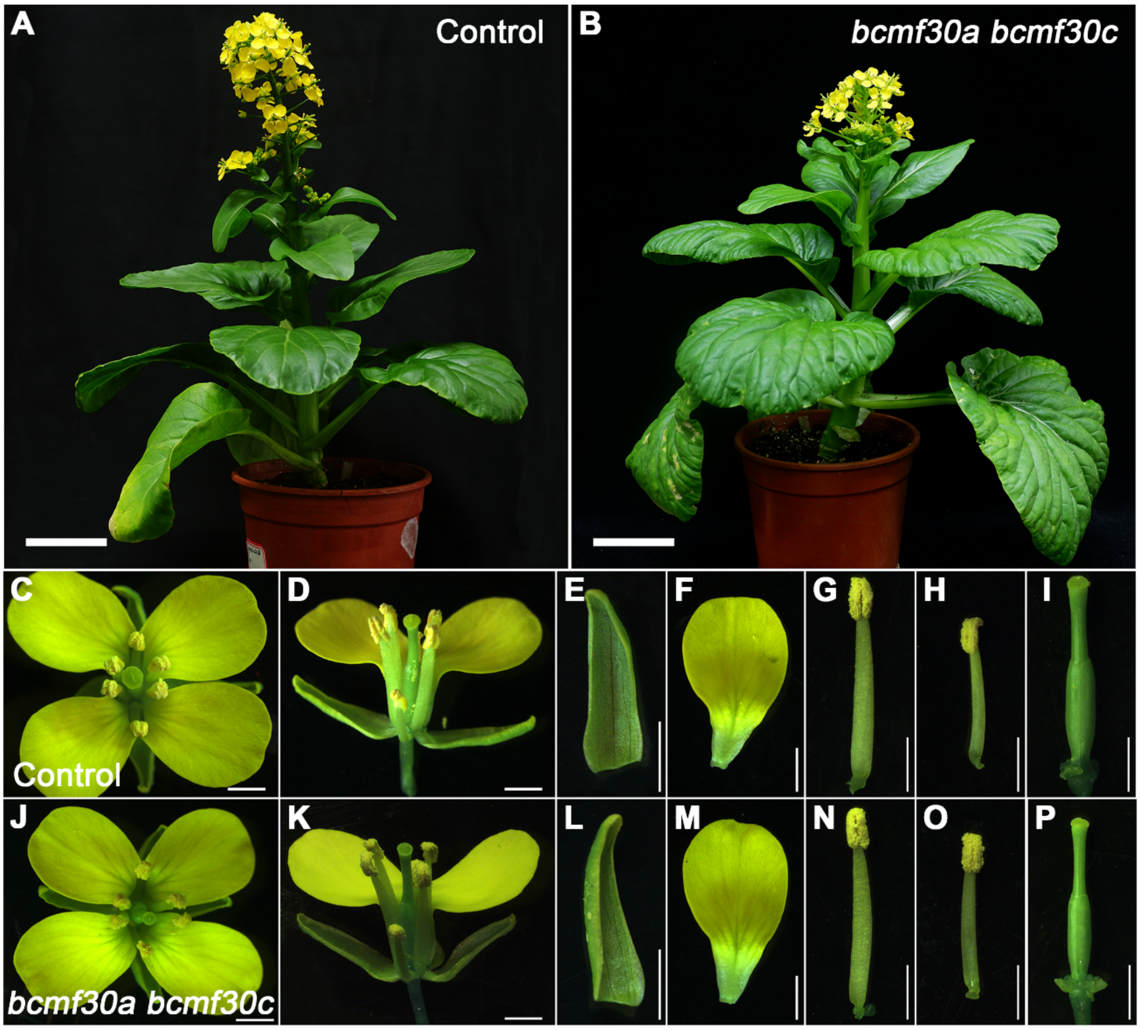

Figure S7. Morphological observation of plants and floral organs of *bcmf30a bcmf30c* plants. (**A**) and (**B**) showed the plants of Control and *bcmf30a bcmf30c*, respectively. (**C**) to (**I**) and (**J**) to (**P**) showed the flower, sepal, petal, stamen and pistil of Control and *bcmf30a bcmf30c*, respectively. Bars = 5 cm in (**A**) and (**B**), and 2 mm in (**C**) to (**P**).
