# Supplementary material for: BcMF30a and BcMF30c, Two Novel Non-Tandem CCCH Zinc-Finger Proteins, Function in Pollen Development and Pollen Germination in Brassica campestris ssp. chinensis
Source: Int J Mol Sci. 2020 Sep 3;21(17):6428. doi: 10.3390/ijms21176428 (PMC7504113; doi:10.3390/ijms21176428)
Supplement: Supplementary file 1 [file ijms-21-06428-s001.zip › Supplementary Figures and Tables/Table S2-S4.pdf]

Table S2 T_0 lines and gene editing of plants transformed by CRISPR-Cas9 vectors.

Target gene	CRISPR-Cas9 Vectors	No. of T_0 lines examined	No. of T_0 lines with mutations in *BcMF30a*	No. of T_0 lines with mutations in *BcMF30c*
BcMF30a	pBI-sgRNA-a	7	0	-
BcMF30c	pBI-sgRNA-c	4	-	0
BcMF30a	pCA-sgRNA-a	5	0	-
BcMF30c	pCA-sgRNA-c	6	-	0
BcMF30a *BcMF30c*	pCA-sgRNA-ac	31	5	5

Table S3 Genotypes of 9 *bcmf30a bcmf30c* plants generated by line ko-41.

T_1 plants	T-DNA insertion	Genotype of BcC3H18a	Genotype of BcC3H18c
ko-41-1	-	C/T	T
ko-41-2	+	C	G/T
ko-41-3	+	C/T	G/T
ko-41-4	+	C/T	G/T
ko-41-5	+	C/T	G/T
ko-41-6	+	C/T	G/T
ko-41-7	-	C/T	T
ko-41-8	+	C/T	T
ko-41-9	+	C/T	G/T

Note: (1) "+" represents T-DNA insertion, "-" represents no T-DNA insertion. (2) "C/T" ("G/T") represents biallelic mutation, i.e., insert C or T (G or T) before the PAM sites of the alleles. "C" ("T") represents homozygous mutations, i.e., C (T) was inserted before the PAM sites of both alleles.

Table S4 Sequence of primers used in this study.

Experiment	Primer Name	Sequence-F(5'-3')	Sequence-R(5'-3')
BcMF30a CDS	BcMF30a-1F/R	ATGAATTTCACAGAATCGAT	CTAGGTAACTGTCGAAATCTC
BcMF30c CDS	BcMF30c-1F/R	ATGAATTTCACAGAATCTATGAAC	CTATGTAACTGTTGAAATCTCCTT
BcMF30a RT-PCR	BcMF30a-2F/R	GTGGATTCGTTGCATAGC	GGGTCCTTGCGTATTTTT
BcMF30c RT-PCR	BcMF30c-2F/R	TGAGGTTGATTCACTCCAGT	GGTCCTACCGTATTTTTCGA
UBC10 RT-PCR	UBC10-1F/R	GGCAACGATAATGGGTCCTAC	CTCCGTGCAGTGGACTCATAC
BcMF30a qRT-PCR	BcMF30a-3F/R	GTGGATTCGTTGCATAGC	TGATCTCCTGCTTGTTCTTAA
BcMF30c qRT-PCR	BcMF30c-3F/R	GTGCAATAATGATGAAGTAGTG	GTTGTTGGATTCTGTTGTGC
UBC10 qRT-PCR	UBC10-2F/R	GGGTCCTACAGACAGTCCTTAC	ATGGAACACCTTCGTCCTAAA
BcMF30a Promoter	BcMF30a-4F/R	AGTTAGTTCCTTAATTTTAGGTATG	CTCCAATAATATCCGTCTTATCA
BcMF30c Promoter	BcMF30c-4F/R	TCCATTTCCCTTCTTATTGT	CTCTAATAATAACGTCTTATCCCA
ProBcMF30a:GUS	BcMF30a-5F/R	TGATTACGCCAAGCTTAGTTAGTTCC TTAATTTTAGGTAT	GACGTAACATCTCCAATAATATCC GTCTTATCA
ProBcMF30c:GUS	BcMF30c-5F/R	TGATTACGCCAAGCTTTCCATTTCCC TTCTTATTGT	GACGTAACATCTCTAATAATAACG TCTTATCCCA
BD-BcMF30a	BcMF30a-6F/R	CATGGAGGCCGAATTCATGAATTTC ACAGAATCGAT	GCAGGTCGACGGATCCCTAGGTAA CTGTCGAAATCTC
BD-BcMF30c	BcMF30c-6F/R	CATGGAGGCCGAATTCATGAATTTC ACAGAATCTATGAAC	GCAGGTCGACGGATCCCTATGTAA CTGTTGAAATCTCCTT
BcMF30a-eGFP	BcMF30a-7F/R	ATTTACAATTACCATGAATTTCACAG AATCGAT	GCCCTTGCTCACCATGGTAACTGTC GAAATCTCCT
BcMF30c-eGFP	BcMF30c-7F/R	ATTTACAATTACCATGAATTTCACAG AATCTATGA	GCCCTTGCTCACCATTGTAACTGTT GAAATCTCCTT
eGFP-BcMF30a	BcMF30a-8F/R	GCTGTACAAGGGATCCATGAATTTC ACAGAATCGAT	TAATTAACTCTCTAGACTAGGTAAC TGTCGAAATCTC
eGFP-BcMF30c	BcMF30c-8F/R	GCTGTACAAGGGATCCATGAATTTC ACAGAATCTATGAAC	TAATTAACTCTCTAGACTATGTAAC TGTTGAAATCTCCTT
sgRNA-a	sgRNA-a-1F/R	GATTGCGTGGAAATACCGGCAGTTG	AAACCAACTGCCGGTATTTCCACG C
sgRNA-c	sgRNA-c-1F/R	GATTGTGATGCAAGAACATGGCGAC	AAACGTCGCCATGTTCTTGCATCAC
sgRNA-ac	sgRNA-ac-1F/R	GATTGAGCGAGCAAGAAGCTTGGTG	AAACCACCAAGCTTCTTGCTCGCTC
CRISPR/Cas9-Seq	Seq-F	TGTAAAACGACGGCCAGT	
CRISPR/Cas9-PCR	PCR-F/R	GATACACCAGACGGAAGAACC	GATGTCGCTCAGCAGGATG
CRISPR/Cas9 T-DNA Insertion	sgRNA-a-2F/R	ACACAGGAAACAGCTATGACC	AAACCAACTGCCGGTATTTCCACG C
	sgRNA-c-2F/R	ACACAGGAAACAGCTATGACC	AAACGTCGCCATGTTCTTGCATCAC
	sgRNA-ac-2F/R	ACACAGGAAACAGCTATGACC	AAACCACCAAGCTTCTTGCTCGCTC
BcMF30a Mutation	BcMF30a-9F/R	CAGAAGAAGTGATAAGACGGA	AAGGCTTGACGAGAACACG
BcMF30a Mutation	BcMF30c-9F/R	GGACTTTTGGGATAAGACGT	TGCTGGGAATGTCAGGTAA
